# A Cataluminescence Sensor Based on NiO Nanoparticles for Sensitive Detection of Acetaldehyde

**DOI:** 10.3390/molecules25051097

**Published:** 2020-03-01

**Authors:** Run-Kun Zhang, Die Wang, Yan-Jun Wu, Yi-Han Hu, Jian-Yu Chen, Jin-Can He, Jing-Xin Wang

**Affiliations:** School of Public Health, Guangdong Pharmaceutical University, Guangzhou 510310, China; mdiewang@163.com (D.W.); 13560295863@163.com (Y.-J.W.); takagihyh@163.com (Y.-H.H.); Ehh117@163.com (J.-Y.C.); hejincan300@163.com (J.-C.H.)

**Keywords:** acetaldehyde, cataluminescence, Ga sensor, NiO

## Abstract

Sensitive and selective detection of harmful gas is an important task in environmental monitoring. In this work, a gas sensor based on cataluminescence (CTL) for detection of acetaldehyde was designed by using nano-NiO as the sensing material. The sensor shows sensitive response to acetaldehyde at a relatively low working temperature of 200 °C. The linear range of CTL intensity versus acetaldehyde concentration is 0.02–2.5 mg/L, with a limit of detection of 0.006 mg/L at a signal-to-noise ratio of three. Mechanism study shows that electronically excited CO_2_ is the excited intermediate for CTL emission during the catalytic oxidation of acetaldehyde on the NiO surface. The proposed sensor has promising application in monitoring acetaldehyde in residential buildings and in the workplace.

## 1. Introduction

Air quality is one of the great concerns in the world. Volatile organic compounds (VOCs) are ubiquitous in the air environment, and the existence of VOCs has greatly deteriorated the air quality [1,2,3,4]. In terms of human health, some VOCs are deemed to be associated with the ill health; some of them are even identified as carcinogens, mutagens, and teratogens [5,6,7,8]. In the modern life, people spend over 90% of their lifetime in indoor environments. Of additional concern, it has been observed that VOCs levels in indoor environments are much higher than the levels in outdoor environments [9,10]. With the continuously growing concern for health care, monitoring of toxic and harmful VOCs in indoor environments has become a requirement in high demand. 

Acetaldehyde is a major environmental pollutant found associated with many human activities, for instance, cigarette smoke, vehicle exhaust, solid biological wastes, and open burning of gas, oil and coal [11,12]. The risk of asthma and cancer will be increased, for people with long-term exposure to acetaldehyde, even at low concentration [13]. Since the concentration of VOCs (including acetaldehyde) found indoors changes frequently every day, every month and every year, and volatile acetaldehyde in an air sample is usually present in trace amounts. Therefore, development of a fast and sensitive method for monitoring of volatile acetaldehyde indoors is urgently needed.

Gas sensors have the advantages of small size, simplicity, low cost and easy operation; they play a crucial role in environment monitoring [14,15,16]. Importantly, they are able to realize real-time monitoring of temporal changes in the concentrations of VOCs. In recent years, there has been a growing interest in the design of gas sensors based on cataluminescence (CTL) for VOCs monitoring, mainly due to CTL-based gas sensors’ many advantages such as low cost, simplicity, high sensitivity and excellent stability. CTL was first reported in 1976 by Breysse et al. [17]; it is a specific kind of chemiluminescence (CL) that is emitted by the catalytic oxidation on the surface of solid catalysts [18,19,20]. In 2002, Zhang’s group first introduced nanomaterial into the design of CTL-based sensors. They found that nanomaterial can remarkably enhance CTL performance, due to the large surface areas, high activity and good adsorbability [21]. After that, a number of CTL-based gas sensors using various nanomaterials were designed for the monitoring of different VOCs [22,23,24,25]. One of the most distinct advantages of using a CTL-based sensor is that the sensing process only consumes the samples and oxygen; the sensing element being a catalyst that is not consumed, and thereby CTL-based sensors possess the capacity for long-term monitoring of an analyte [26,27].

Synthesis and screening of appropriate nanomaterial is the key to design of a CTL-based sensor. Herein, nanomaterial NiO was synthesized by a direct precipitation method. We found that the as-prepared NiO showed high sensitivity and selectivity to acetaldehyde. A novel CTL-based sensor for acetaldehyde was designed. *N*-propanal, *n*-butanal, acetone etc. could not produce response when their concentrations fell below 0.05 mg/L. Compared with the previous CTL-based sensors for acetaldehyde, the proposed sensor is able to detect a wider range of acetaldehyde (0.02–2.5 mg/L), and the optimal working temperature of the present sensor is about 25 to 30 centigrade lower than the best results obtained by the published works [28,29].

## 2. Results and Discussion 

### 2.1. Characterization of Nickel Oxide Nanoparticles

**Figure 1:**
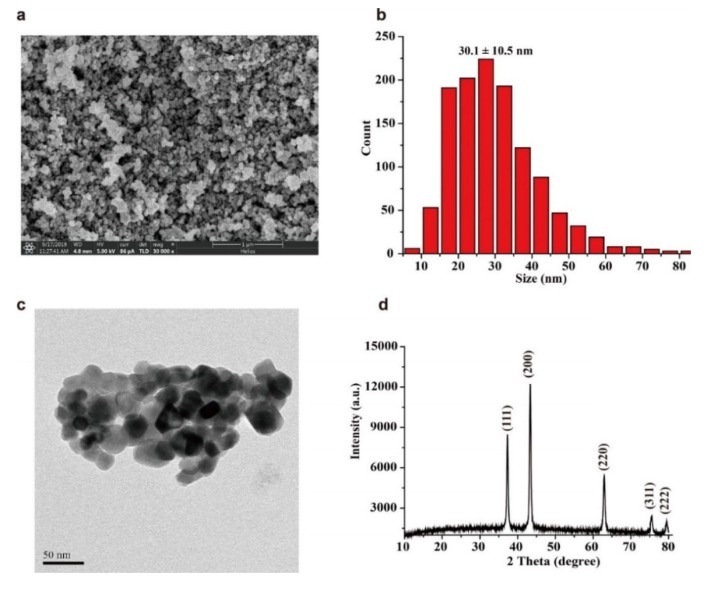
The SEM image (**a**), the particle size distribution histogram (**b**), the TEM image (**c**) and XRD pattern (**d**) of the as-prepared NiO.

The characterization results of the as-prepared NiO are shown in Figure 1. A typical SEM image of the as-prepared NiO is shown in Figure 1a. ImageJ software was used to evaluate the diameter of nanoparticles. The statistical size distribution histograms obtained from the SEM image is shown in Figure 1b. The particle sizes of the NiO lie in the range of 8.2 to 82.7 nm, with an average diameter of 30.1 ± 10.5 nm. The TEM image in Figure 1c shows that as-prepared NiO nanoparticles reveal both spherical and irregular particle shape. Figure 1d shows the XRD pattern of the as-prepared NiO. All the diffraction peaks in the pattern can be indexed as cubic NiO phase (JCPDS 47–1049), The peaks at 2*θ* = 37.3, 43.3, 62.9, 75.5 and 79.5° in the XRD pattern can be readily assigned to (111), (200), (220), (311) and (222) crystal planes, respectively. The sharpness and the intensity of the peaks indicate that the prepared NiO possesses good crystalline nature.

### 2.2. Selectivity

**Figure 2:**
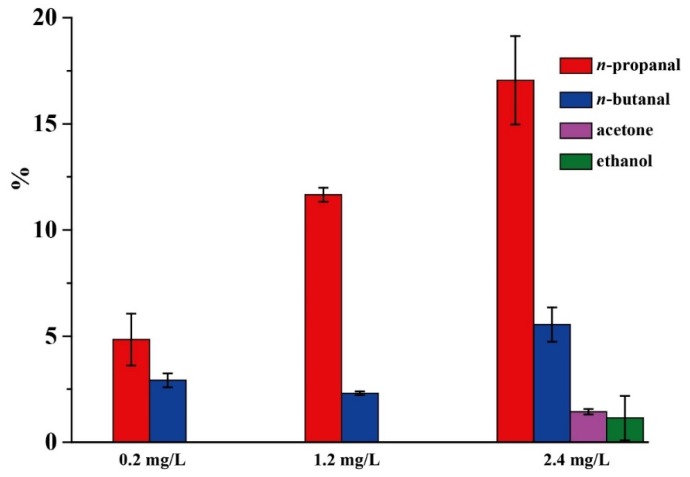
The percentage of cataluminescence (CTL) responses of different compounds at different concentrations compared to acetaldehyde. Air flow rate: 80 mL/min, temperature: 200 °C, wavelength: 425 nm. Error bars stand for ± S.D. (standard deviation).

Selectivity is one of the most important performance criteria that should be first considered to design the sensor, as the selectivity will directly affect the accuracy. Selectivity of a sensor is usually defined as the ratio of the response of an interfering analyte to that of a target analyte. Thus, the selectivity of a CTL-based sensor can be expressed as [30]:

(1) $$\mathrm{Selectivity}\;\left(\%\right)=\frac{S_{other\;analyte}}{S_{target\;analyte}}\times 100\%,\;$$

where *S* stands for the relative CTL intensity, and *S* is equal to the apparent CTL intensity (the peak value directly recorded by the instrument) minus the background noise (*N*). The prepared nano-NiO was used for CTL sensing of different compounds to investigate its selectivity. Results found that when exposed to 0.05 mg/L of acetaldehyde, *n*-propanal, *n*-butanal, acetone, ethanol, methanol, formaldehyde, benzene, toluene, *o*-xylene, *m*-xylene, *p*-xylene, *n*-hexane, acetic acid, ethyl acetate, ammonia and carbon dioxide, only acetaldehyde produced a CTL response. Subsequently, 0.2, 1.2 and 2.4 mg/L of these compounds were further tested. As shown in Figure 2, when the tested concentration increases to 0.2 mg/L, *n*-propanal and *n*-butanal begin to produce CTL responses, which are about 4.9% and 2.9% of the CTL response of the same concentrations of acetaldehyde, respectively. The CTL responses of *n*-propanal and *n*-butanal, at 1.2 mg/L, are about 11.6% and 4.3% of the CTL response of the same concentrations of acetaldehyde, respectively. When the tested concentration increases to 2.4 mg/L, CTL responses of *n*-propanal, *n*-butanal, acetone and ethanol were observed, while other compounds still did not produce CTL responses. The intensities of *n*-propanal and *n*-butanal, acetone, and ethanol are 17.5%, 8.5%, 1.4% and 1.1% of the intensity of acetaldehyde, respectively. CTL responses of other compounds are relatively lower even when they are at higher concentrations, indicating that the sensor has a good selectivity to acetaldehyde, and thereby the prepared nano-NiO is a good candidate for designing CTL-based sensors for acetaldehyde.

### 2.3. Effect of Detecting Wavelength

**Figure 3:**
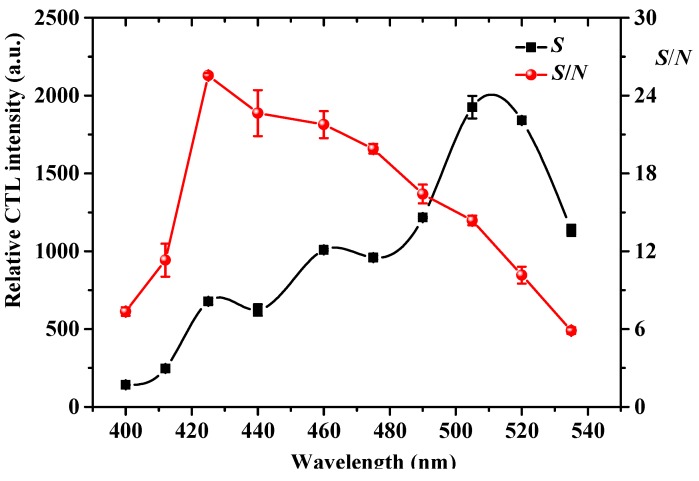
The effect of detecting wavelength on the relative CTL intensity and signal-to-noise ratio (*S*/*N)*. Temperature: 200 °C, air flow rate: 80 mL/min, concentration: 1.2 mg/L. Error bars stand for ±S.D.

The effect of detecting wavelength on CTL sensing of acetaldehyde was investigated by using a series of optical filters to record the relative CTL intensity (*S*) and signal-to-noise ratio (*S*/*N*). The result is shown in Figure 3. Three peaks at 425, 460 and 505 nm are observed on the CTL emission spectrum of acetaldehyde on the nano-NiO surface. Although the maximum emission wavelength is 505 nm, the infrared background radiation increases with the wavelength, leading to a decrease in *S*/*N* at long wavelengths. Since the maximum *S*/*N* is obtained at 425 nm, we used 425 nm as the wavelength for detecting acetaldehyde.

### 2.4. Effect of Working Temperature

**Figure 4:**
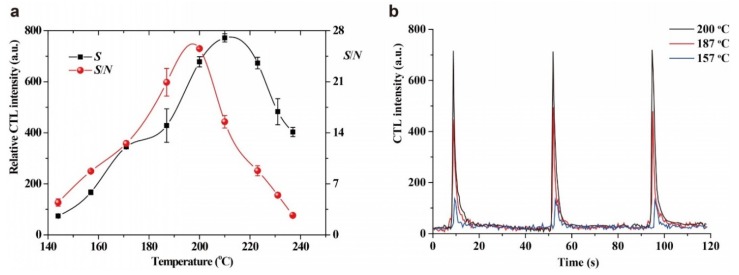
(**a**) Effect of temperature on the relative CTL intensity and the *S*/*N*. Test conditions were: air flow rate: 80 mL/min. wavelength: 425 nm, concentration:, 1.2 mg/L. (**b**) The CTL response profiles of acetaldehyde at working temperatures of 157 °C, 187 °C and 200 °C. Test conditions were: air flow rate: 80 mL/min, wavelength: 425 nm, concentration: 1.2 mg/L. Error bars stand for ± S.D.

The effect of working temperature on the CTL sensing of acetaldehyde was investigated. As shown in Figure 4a, the relative CTL intensity increases with working temperature before reaching 210 °C, and then decreases. Possibly the increase in working temperature results in increasing reaction rate, but quenching of CTL intensity easily occurs at higher temperature because of the accelerated molecular motion. Note that *S*/*N* increases with working temperature before 200 °C and then decreases, which may be attributed to the stronger infrared background radiation emitted at higher working temperature. Therefore, a working temperature of 200 °C was used for detection of acetaldehyde. It is worth mentioning that the working temperature of the present sensor is much lower than that of most previous CTL-based sensors (usually above 250 °C or higher) [27]. The relatively lower energy consumption is favorable towards its application in the future.

Figure 4b shows the CTL response profiles of acetaldehyde at different working temperatures. We found that the CTL signals reach their maximum values within 3 s after sample injection, with the recovery time within 10 s, indicating the fast-response and rapid-recovery of the sensor. An increase in working temperature has almost no influence on the response time and on the recovery time, which indirectly proves that the oxidation of acetaldehyde on the nano-NiO surface occurs very rapidly, even at relatively lower working temperatures.

### 2.5. Effect of the Air Flow Rate

**Figure 5:**
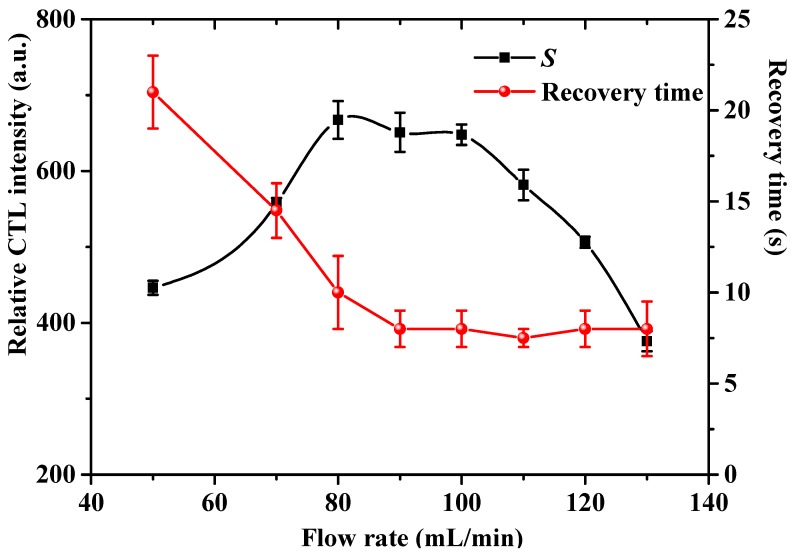
Effect of air flow rate on the relative CTL intensity and recovery time. Test conditions were: temperature: 200 °C, wavelength: 425 nm, concentration: 1.2 mg/L. Error bars stand for ± S.D.

The effect of the air flow rate on the CTL sensing of acetaldehyde was investigated. Figure 5 shows the change trends of the relative CTL intensity and recovery time versus air flow rate. The relative CTL intensity increases before 80 mL/min, and then decreases with increasing flow rate. We found that the relative CTL intensity increases first before 80 mL/min, and then decreases gradually with an increase of flow rate. The recovery time also increases with an increase of flow rate before 80 mL/min. Noteworthy, it seems that the flow rate has no influence on the recovery time in the range of 80–130 mL/min. The above results indicate that under low flow rate, the total reaction rate of the oxidation of acetaldehyde is controlled by the diffusion rate, and thereby the relative CTL intensity increases with increasing flow rate, and the recovery time shows a reverse trend. However, the total reaction rate is dependent on the oxidation rate under higher flow rate, as the acetaldehyde molecules leave the catalyst surface rapidly due to the driving force of the higher flow rate, rendering a decrease in the relative CTL intensity for the insufficient reaction. Since the total reaction rate is controlled by the oxidation rate, further increase in flow rate has almost no influence on the recovery time. We used an 80 mL/min flow rate for detection of acetaldehyde due to observing the highest CTL response at this flow rate.

### 2.6. Analytical Characteristics

**Figure 6:**
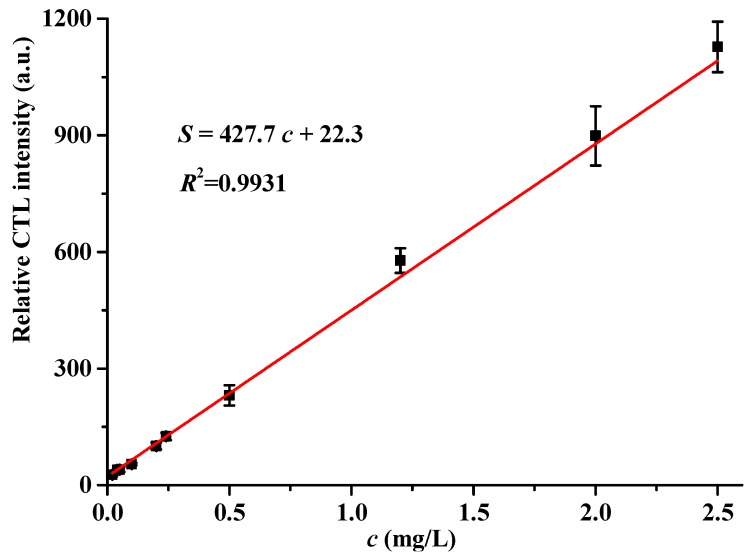
The calibration curve between the relative CTL intensity and the concentration of acetaldehyde. Test conditions were: air flow rate: 80 mL/min, temperature: 200 °C, wavelength: 425 nm. Error bars stand for ± S.D.

Under the above optimized conditions, the relative CTL intensity is linearly related to the concentration of acetaldehyde within the range of 0.02–2.5 mg/L. As shown in Figure 6, the linear regression equation is Y = 427.7X + 22.3, where Y is the relative CTL intensity, and X is the concentration of acetaldehyde. The correlation coefficient is 0.9931, and the limit of detection (LOD) is 0.006 mg/L (*S*/*N* = 3). The permitted long-term exposure limit and short-term exposure limit of acetaldehyde in the workplace are 0.037 and 0.092 mg/L (EH40/2005, England), respectively [31]. The limit of detection of the sensor is lower than this threshold limit value, showing the promising application of the present sensor in air quality monitoring.

Table 1 compares the performances of the present sensor with other sensors reported in the literature. Compared with the CTL sensors based on BaCO_3_ and Zeolite, the present sensor shows advantages of lower working temperature and shorter recovery time. The working temperature, recovery time and LOD of the present sensor are superior to the performances of the electrochemistry sensors based on In_2_O_3_.

### 2.7. Sample Analysis

*N*-propanal and *n*-butanal are the two main interferents for the detection of acetaldehyde by the present sensor; ethanol is used for synthesis of acetaldehyde in the industry; *n*-hexane and ethyl acetate are two commonly used organic solvents; and formaldehyde, benzene, acetic acid are common air pollutants. These compounds are likely to co-exist with acetaldehyde in polluted air. Five artificial air samples containing different compositions were prepared, and then were analyzed by the proposed sensor. As shown in Table 2, sample 1 is a mixture of acetaldehyde and *n*-propanal; sample 2 is made up of acetaldehyde and *n*-butanal; sample 3 was prepared by mixing acetaldehyde with ethanol; sample 4 consists of acetaldehyde, *n*-hexane and ethyl acetate; sample 5 is composed of formaldehyde, benzene and acetic acid. The acetaldehyde in the five artificial samples was well quantified with satisfactory recoveries ranging from 96.0% to 116.0%, indicating the promising application of the sensor for sensing acetaldehyde in residential building and in the workplace.

### 2.8. Sensing Mechanism

It was reported that the oxygen in the air (carrier gas) can be chemisorbed to form active adsorbed oxygen species (O_2_^−^, O^−^, and O^2^^−^) on the heated surface of NiO [33,34]. It was found that the amount of O^−^ species increases with the increase of temperature until 500–550 °C, and it is the predominant species up to 150–200 °C [33]. Because the optimal working temperature of the present sensor is 200 °C, the O^−^ species was considered as the main active adsorbed oxygen species for oxidation of acetaldehyde. In order to further explore the sensing mechanism of the proposed CTL sensor based on NiO, the products from the catalytic oxidation of acetaldehyde on the NiO surface were analyzed by a gas chromatography instrument equipped with a flame ionization detection and methane reformer. Acetic acid and carbon dioxide were identified as the main reaction products, which is in accordance with a previous report revealing that acetaldehyde can be oxidized into acetic acid by active oxygen species, and then acetic acid is able to further oxidize into CO_2_ [35,36]. It is now widely believed that CTL emission requires production of excited intermediates during the catalytic oxidation process [37]. The CTL emission spectrum (Figure 2) of acetaldehyde on a nano-NiO surface consists of a broad band with a maximum at about 505 nm, which is similar to the emission spectrum from electronically excited CO_2_ (CO_2_*) [24,38]. Therefore, we speculated that CO_2_* is the possible excited intermediate for CTL emission during the catalytic oxidation process of acetaldehyde on a NiO surface. The possible mechanism could be described as follows:
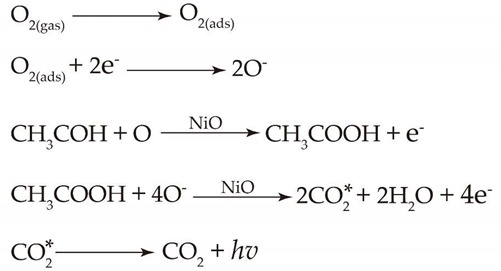


## 3. Experimental Section

### 3.1. Materials and Reagent

Chloride hexahydrate (NiCl_2_·6H_2_O), aqueous ammonia, acetaldehyde, *n*-propanal, *n*-butanal, acetone, ethanol, methanol, formaldehyde, benzene, toluene, *o*-xylene, *m*-xylene, *p*-xylene, *n*-hexane, acetic acid and ethyl acetate were purchased from Aladdin Reagent Co. Ltd. (Shanghai, China). Carbon dioxide was purchased from The National Standard of Material Resources Network (Beijing, China).

### 3.2. Instrumentation

A BPCL ultra-weak luminescence analyzer equipped with a photomultiplier detector (Guangzhou Microphotonics Technologies Co., Ltd., Guangzhou, China) was used to detect and record the CTL signal. A gas chromatography instrumentation equipped with a flame ionization detector detector and methane reformer (Shanghai Chromatographic instrument Co., Ltd., Shanghai, China) was used to detect the reaction products. The morphology and particle size were examined by a scanning electron microscope (Helios G4 CX) and transmission electron microscopy (FEI Tecnai G2 F20). Powder X-ray diffraction data were collected on a Rigaku Ultima IV X-Ray Diffractometer using CuKα radiation with a scan speed of 5°/min ranging from 10° to 80°. (λ = 1.54 A°, operated at 40 mA and 40 kV).

### 3.3. Synthesis of Nickel Oxide Nanoparticles

First, 1.0 g of NiCl_2_·6H_2_O was dissolved into 50 mL of distilled water, then 4 mL aqueous ammonia (25%) was added to the above solution with vigorous stirring. The mixture was stirred constantly for 30 min, the resulting suspension was transferred into an autoclave and then oven-dried at 80 °C. Finally, the oven-dried precipitates were annealed at 350 °C in a furnace in air for 2.5 h.

### 3.4. Fabrication of the CTL Sensor

**Figure 7:**
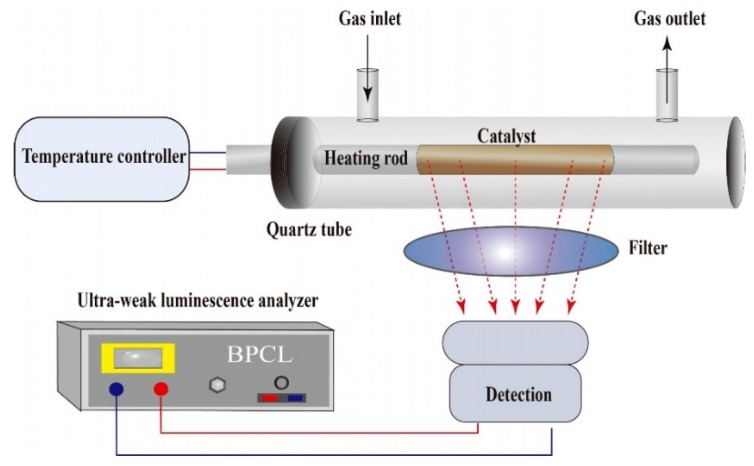
Schematic diagram of the CTL sensor for acetaldehyde.

The schematic diagram of the CTL sensor for acetaldehyde is shown in Figure 7. The as-prepared 0.8 g NiO was mixed with 3.0 mL deionized water to obtain a pulp suspension. Subsequently, a certain volume of pulp suspension was dripped onto the surface of a heating rod and then was annealed in air to form a catalyst layer. The heating rod was placed into a home-made quartz tube with a gas inlet and gas outlet. The working temperature of the heating rod was controlled by a temperature controller via tuning the output voltage. A mini-airpump was used to support air flow and to transport the sample. Once the sample was transported to the catalyst layer, it was oxidizedby the oxygen in the air to produce a CTL emission. The CTL signal was detected and recorded by the BPCL Ultra-weak Luminescence Analyzer. The detecting wavelength could be changed by choosing the optical filters.

## 4. Conclusions

In conclusion, a CTL-based sensor for acetaldehyde with the advantages of high selectivity, fast response and rapid recovery was designed by using nano-NiO. Compared with other CTL-based sensors for acetaldehyde, the most distinctive advantage of the proposed sensor is that it can sense acetaldehyde at relatively low working temperatures. The sensor was successfully applied to detect acetaldehyde in artificial air samples. Mechanism study led us to describe a possible mechanism by which electronically excited CO_2_ is the excited intermediate for CTL emission during the catalytic oxidation process of acetaldehyde on the NiO surface. This work provides a simple and rapid method for sensitive sensing of acetaldehyde in the field of air quality monitoring.

## Figures and Tables

**Table 1:** Comparison of performances of acetaldehyde gas sensors.

Principle	Materials	Temperature (°C)	Recovery Time (s)	LOD (mg/L)	References
CTL	NiO	200	10	0.006	Present work
CTL	BaCO_3_	225	50	0.001	[28]
CTL	Zeolite	230	~100	0.02	[29]
Electrochemistry	In_2_O_3_	300	480	0.002	[32]

**Table 2:** Detection of acetaldehyde in five artificial samples by the present sensor (*n* = 5).

Sample No.	Mixture	Spiked Values (mg/L)	Measured Values (mg/L)	Recovery (%)
1	Acetaldehyde	0.5	0.57 ± 0.01	114.0 ± 2.0
	*n*-Propanal	2.0		
2	Acetaldehyde	0.5	0.58 ± 0.04	116.0 ± 8.0
	*n*-Butanal	2.0		
3	Acetaldehyde	0.5	0.48 ± 0.01	96.0 ± 2.0
	Ethanol	2.0		
4	Acetaldehyde	0.5	0.52 ± 0.02	104.0 ± 4.0
	*n*-Hexane	2.0		
	Ethyl acetate	2.0		
5	Acetaldehyde	0.5	0.52 ± 0.03	104.0 ± 6.0
	Formaldehyde	2.0		
	Benzene	2.0		
	Acetic acid	2.0		
